# Preparation of Quantum Dot-Embedded Photonic Crystal Hydrogel and Its Application as Fluorescence Sensor for the Detection of Nitrite

**DOI:** 10.3390/nano11113126

**Published:** 2021-11-19

**Authors:** Rongzhen Li, Lian Li, Bin Wang, Liping Yu

**Affiliations:** 1 Department of Chemistry, School of Science, Tianjin University, Tianjin 300350, China; lirongzhen@tju.edu.cn; 2 Department of Chemistry, School of Science, Tianjin Chengjian University, Tianjin 300384, China; zlarina90@outlook.com (L.L.); wb1988@tju.edu.cn (B.W.)

**Keywords:** fluorescent hydrogel, quantum dots, photonic crystal, sensor

## Abstract

The development of fluorescence sensing platforms with excellent photoluminescence capabilities is of great importance for their further application. In this work, a photonic crystal structure was successfully applied to enhance the luminescence performance of fluorescent hydrogel, and the application of the obtained hydrogel as a fluorescence sensor was explored. A polystyrene photonic crystal template was constructed via vertical deposition self-assembly; then, the precursor solution containing polyethylenimine-capped CdS quantum dots (PEI-CdS QDs) and monomers filled in the gap of the template. After the polymerization process, the desired hydrogel was obtained. PEI-CdS QDs endowed the hydrogel with its fluorescence property, while interestingly, the photonic crystal structure showed a significant enhancement effect on the fluorescence-emission capability. The mechanism of this phenomenon was revealed. Moreover, this hydrogel could be used as a reusable fluorescence sensor for the detection of nitrite in water with good selectivity. The limit of detection was determined to be 0.25 μmol/L, which is much lower than the maximum limit for nitrite in drinking water.

## 1. Introduction

Since the concept of colloidal quantum dots (QDs) was first proposed by Bell Lab scientist Brus in 1983, extensive scientific research has been conducted, and remarkable progress has been made on this hot topic [1,2,3,4]. Various QDs, including semiconductor nanocrystalline QDs [5,6,7], carbon QDs [8,9], graphene QDs [10,11] and polymer QDs [12,13], have been reported in the past two decades. These QDs are usually quasi-spherical particles with diameters ranging from 1 nm to 15 nm. Ascribed to quantum confinement and edge effects, QDs exhibit fascinating fluorescence properties [14,15]. Compared with other traditional fluorescent materials, QDs possess various advantages, such as a low production cost, easy preparation, tunable photoluminescence, good stability and biocompatibility, and high quantum yields, which make them promising fluorescent materials in many fields of application [16,17], especially as fluorescent probes in sensing analysis [18,19].

QDs could show excellent photoluminescence properties when well dissolved in solvent. However, when they are in a higher concentration or in a solid state, their fluorescence is affected by an aggregation-induced quenching effect and significantly weakened [20]. Thus, most QD fluorescence-sensing assays are carried out in solution, especially in water [21,22]. Though numerous sensing methods have been achieved via a liquid platform of QD fluorescence systems, there are still some drawbacks, which need to be fixed. Liquid platforms always suffer from a shorter shelf life and often require scientific intervention. Moreover, they are difficult to implement in field-based settings [23]. Therefore, developing novel sensing systems to solve these problems is highly desired.

Hydrogels are three-dimensional cross-linked hydrophilic polymer networks, and their capacity to adsorb water-soluble substances makes them particularly useful for promoting interactions between ionic compounds and functional sites in the matrix [24,25]. In addition, their potential to incorporate various nanomaterials into the matrix could further enrich their functions [26,27,28]. Recently, some research groups embedded QDs into hydrogel matrices to produce fluorescent hydrogels and explored their applications in analytical sensing [29,30,31]. Truskewycz incorporated carbon quantum dots into a ZnO/PVP hydrogel matrix. Their obtained fluorescent hydrogel could specifically interact with Cr^6+^, and there was a good linear relationship between fluorescence intensity and Cr^6+^ concentration [23]. Yan prepared a QD-based chitosan hydrogel and achieved the sensing of Hg^2+^ with good selectivity [32]. Moreover, the sensing of some other organic compounds, such as progesterone [33], glucose [34], and even the avian influenza virus [35], has also been achieved by using QD-embedded hydrogels. QD-embedded hydrogels have become a promising system with which to replace traditional liquid and solid fluorescence-sensing platforms.

Herein, we fabricated polyethylenimine-capped CdS quantum dots (PEI-CdS QDs) and embedded them into a polyacrylamide copolymer hydrogel with a photonic crystal structure. PEI-CdS QDs endowed the hydrogel with fluorescence properties, while, interestingly, the photonic crystal structure exhibited a significant enhancement effect on the fluorescence of the hydrogel, which improved the application prospects of the material. The obtained quantum dot-embedded photonic crystal hydrogel could work as a fluorescence sensor for the detection of nitrite in water with good selectivity and high sensitivity. Importantly, the quantum dot-embedded photonic crystal hydrogel could be reused after rinsing it with distilled water after the sensing process.

## 2. Experimental Section

### 2.1. Materials

Acrylamide (AMD) was purchased from Tianjin Kermel Chemical Company (Tianjin, China). *N*,*N*′-methylene-bis-acrylamide (BIS) was supplied by Tianjin Fuchen Chemical Reagents Factory (Tianjin, China). 2,2′-Azobis(2-methylpropionamidine) dihydrochloride (AIBA) was provided by Tianjin Heowns Biochem Company (Tianjin, China). Polyethyleneimine (PEI) was purchased from Aldrich (Shanghai, China). CdCl_2_•2.5H_2_O and Na_2_S•9H_2_O were purchased from Tianjin Yuanli Chemical Company (Tianjin, China). AMD and BIS were purified before use. All the reagents used in the experiment are analytically pure.

### 2.2. Characterization

JEM-2100F transmission electron microscopy (TEM) (Jeol, Tokyo, Japan) was used to record the morphologies of PEI-CdS QDs. The surface morphologies of hydrogel films were characterized by Regulus 8100 field-emission scanning electron microscopy (SEM) (Hitachi, Tokyo, Japan). A F-320 fluorescence spectrophotometer was utilized to record the fluorescence spectra. The UV-Vis diffuse-reflectance spectra were recorded on a TU-1901 spectrophotometer (Persee, Beijing, China), and the UV-Vis absorption spectra were recorded on a UV-1800PC spectrophotometer (Mapada, Shanghai, China). The reflectance spectra of photonic crystal were measured by a 380–1050 nm fiber optic spectrometer (Jinke JKHQ-D1, Tianjin, China).

### 2.3. Synthesis of PEI-CdS QDs

The method of synthesizing PEI-CdS QDs was followed from our previous work [36]. Briefly, hyperbranched PEI was used as the functional polymer, and 0.648 mL CdCl_2_ aqueous solution was added into PEI aqueous solution. Then, 6.480 mL of Na_2_S aqueous solution was dropped into the solution. After 1 h, extra CdCl_2_ aqueous solution was added into the system again and stirred for a certain time. The obtained PEI-CdS QDs were stored in the dark at a concentration of 10 g/L after freeze drying.

### 2.4. Preparation of Photonic Crystal Templates

Photonic crystal templates were prepared using the vertical-deposition self-assembly method as follows: 1.2 g of polystyrene microsphere emulsion and 50 mL of distilled water were mixed in a beaker, then glass slides were inserted vertically into the emulsion. The beaker was put in a water bath (43 °C). After the continuous evaporation of water, polystyrene microspheres were deposited layer by layer on the glass slides to form photonic crystal templates, and the templates on the glass slides were dried in an oven at 80 °C before use.

### 2.5. Preparation of QD-Embedded Photonic Crystal Hydrogel

In this step, 2 g acrylamide, 0.045 g BIS and 0.024 g AIBA were dissolved in the prepared PEI-CdS QDs solution to obtain the precursor solution. The “sandwich” method was used to cover a glass slide on the photonic crystal template, and the precursor solution was dropped into the gap between the two glass slides. Via the capillary force, the precursor liquid gradually filled into the entire gap. Then, the precursor solution was polymerized together with PEI-CdS QDs in a photonic crystal template after heating at 60 °C for 5 h. Finally, PEI-CdS QD-embedded photonic crystal hydrogel was obtained when the polymer film was carefully torn off from the slide. The control fluorescent hydrogel films were prepared with the same method, using two glass slides without the photonic crystal structure.

### 2.6. Sensing of Nitrite

A 0.01 mol/L nitrite solution was prepared, diluted to different concentrations (10^−6^ mol/L–10^−3^ mol/L), and stored in the refrigerator before further use. Amounts of 10 μL of nitrite solutions at different concentrations were added to phosphate-buffered solution, and the final concentration of nitrite ranged from 10^−8^ mol/L to 10^−5^ mol/L. The hydrogel was immersed in the nitrite solution for 10 min; then, the water on the hydrogel surface was gently sucked up using filter paper, and the fluorescence spectrum was recorded subsequently. The fluorescence intensity of the films soaked in buffer solution was denoted as blank value F_0_, and the fluorescence intensity measured after soaking in nitrite solution was denoted as F.

Solutions of 0.01 mol/L NaNO_2_, KNO_3_, Na_2_CO_3_, NaHCO_3_, Na_2_SO_4_, Na_2_SO_3_, CH_3_COONa, Na_3_PO_4_, NaCl, KBr, NaI, and Na_2_S were prepared for selectivity investigations. The hydrogel film was immersed in the above various anion solutions for 10 min, and the fluorescence emission spectrum was measured.

For the recovery test, the hydrogel film was placed in distilled water for 10 min after one measurement in nitrite solution. Then, the fluorescence spectrum was recorded, and the hydrogel was immersed in nitrite solution again to commence the next cycle.

## 3. Results and Discussion

### 3.1. Preparation of QD-Embedded Photonic Crystal Hydrogel

**Figure 1:**
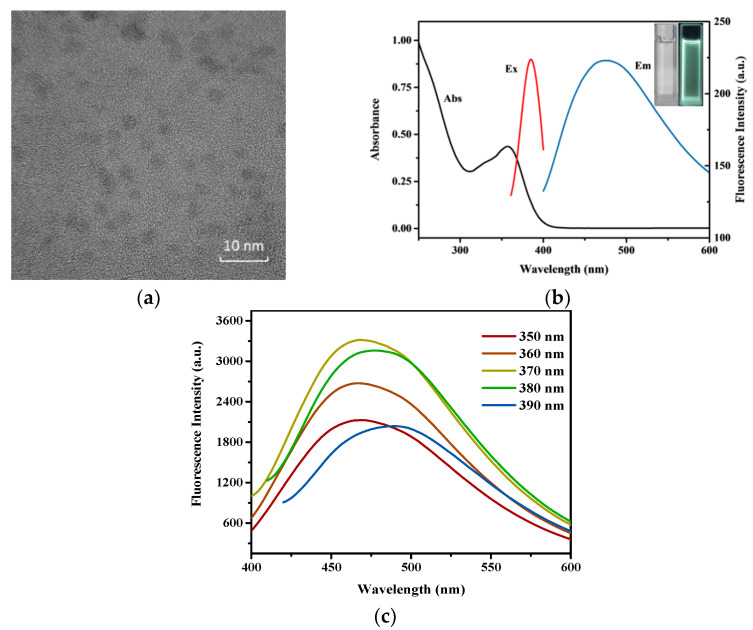
TEM image (**a**), UV-Vis absorption and the optimal excitation and emission wavelengths (**b**) and the excitation-dependent fluorescence spectra from 350 nm to 390 nm (**c**) of PEI-CdS QDs.

PEI-CdS QDs were synthesized by an environmentally friendly, one-pot aqueous-phase synthesis method according to our previous work [36]. First, CdCl_2_ solution was mixed with PEI with gentle stirring, then Na_2_S aqueous solution was dropped into the mixture. After the growth of CdS nanocrystals for a certain amount of time, extra Cd^2+^ was added to cap the formed quantum dots. The obtained PEI-CdS QDs were characterized by TEM (Figure 1a). It can be seen that PEI-CdS QDs are quasi-spherical particles with diameters ranging from 3 nm to 7 nm, and their average particle size is ca. 5 nm. The optical properties of PEI-CdS QDs were studied. A strong UV-Vis adsorption peak at 355 nm was found in the UV-Vis spectrum, which is in accordance with our previous research. PEI-CdS QDs aqueous solution could emit bright-blue fluorescence under the illumination of UV light (inset of Figure 1b). In the fluorescence spectra, the optimal excitation and emission wavelengths centered at 370 nm and 475 nm, respectively. Figure 1c shows the excitation-dependent fluorescence spectra of PEI-CdS QDs. It was found that when the excitation wavelengths change from 350 nm to 390 nm, the emission peak only shows a slight red shifting. In addition, the quantum yield of PEI-CdS QDs was calculated to be 7.5% by using quinine sulfate as the standard.

**Scheme 1:**
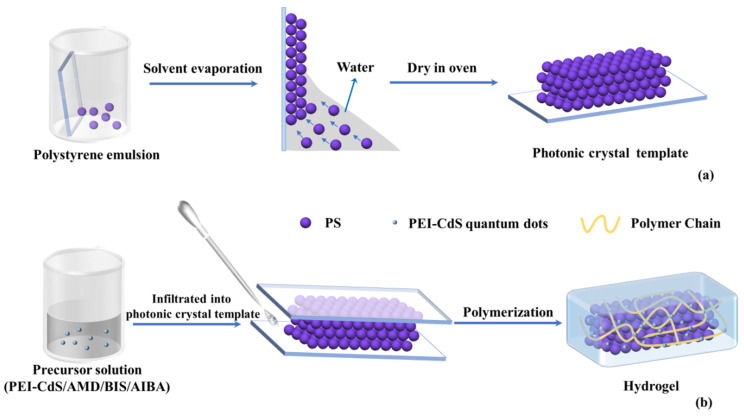
Schematic diagram of the PEI-CdS QD-embedded photonic crystal hydrogel preparation process. (**a**) Preparation of photonic crystal template, (**b**) preparation of QD-embedded photonic crystal hydrogel.

The PEI-CdS QD-embedded photonic crystal hydrogel was fabricated via a two-step procedure (Scheme 1). First, the photonic crystal template was constructed on the surface of a slide via the vertical deposition self-assembly of polystyrene microspheres. Then, the precursor solution containing AMD, BIS, AIBA, and PEI-CdS QDs filled in the gaps of the photonic crystal template, which was heated at 60 °C for 5 h to promote polymer formation. After these processes, the obtained hydrogel film was carefully removed from the slide.

**Figure 2:**
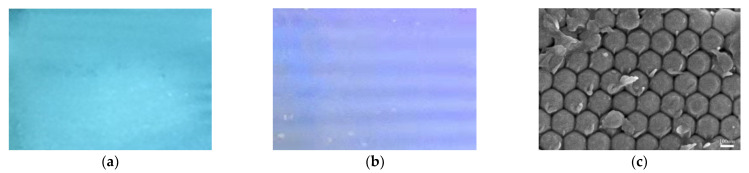
The photo under UV light (**a**) and visible light (**b**) and the SEM image (**c**) of the PEI-CdS QD-embedded photonic crystal hydrogel.

This hydrogel film shows bright blue-green fluorescence when irradiated under UV light (Figure 2a), indicating that it efficiently inherits the fluorescent properties of PEI-CdS QDs. Figure 2b shows the photo of the hydrogel film under visible light. A distinct blue structure color was observed, which demonstrates that the prepared hydrogel film has a photonic crystal structure. In addition, the SEM image in Figure 2c further reveals the photonic crystal structure in the hydrogel.

**Figure 3:**
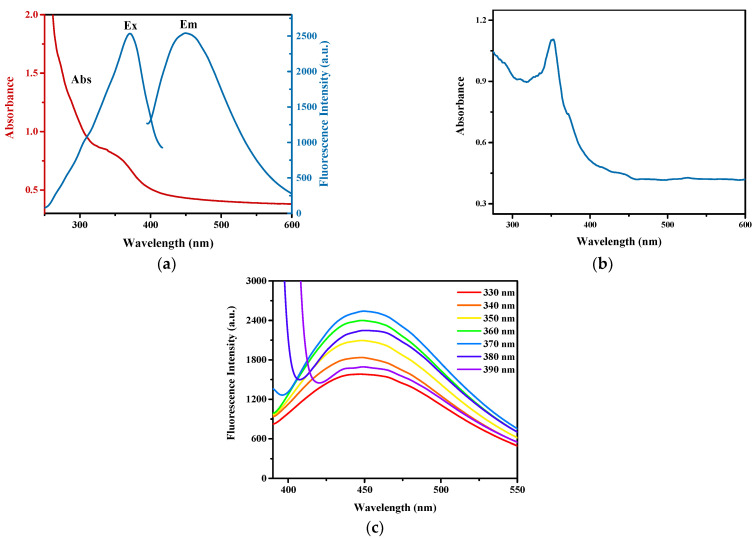
UV-Vis absorption and the optimal excitation and emission wavelengths (**a**), UV-Vis diffuse reflectance spectra (**b**), and excitation-dependent fluorescence spectra from 330 nm to 390 nm (**c**) of the PEI-CdS QD-embedded photonic crystal hydrogel.

The optical properties of the obtained hydrogel were measured and are shown in Figure 3. In the UV-Vis spectrum, an adsorption shoulder peak around 355 nm was found. In the fluorescence spectra, the optimal excitation and emission wavelengths centered at 380 nm and 478 nm, respectively (Figure 3a). As shown in Figure 3b, there was also a narrow peak at 352 nm in the UV-Vis diffuse reflectance spectra, which is consistent with the absorption peak of the QD-embedded photonic crystal hydrogel. Figure 3c shows the excitation-dependent fluorescence spectra of the hydrogel. It was found that when adjusting the excitation wavelength from 330 nm to 390 nm, the fluorescence intensity increased at first and then decreased, while the position of the emission peak hardly moved. In the present work, 370 nm was chosen as the excitation wavelength according to the fluorescence intensity of the hydrogel.

### 3.2. Optimization of the Fluorescence Property of the Hydrogel

**Figure 4:**
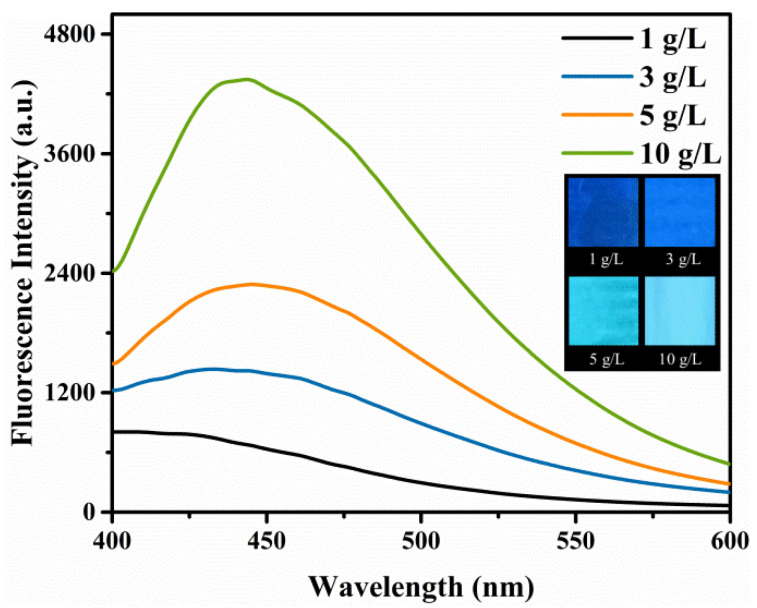
Fluorescence emission spectra of the hydrogel with different embedded PEI-CdS QD contents in the range of 1 g/L to 10 g/L.

In order to optimize the fluorescence emission capability of the hydrogel, the content of PEI-CdS QDs in the hydrogel was first studied. A series of precursor solutions containing different amounts of PEI-CdS QDs were used during the polymerization process, and the fluorescence emission spectra of these obtained hydrogels were measured, as shown in Figure 4. With the increase in PEI-CdS QD concentration, the fluorescence intensity of the obtained hydrogel increased correspondingly. No aggregation-induced quenching was observed even when the concentration of the QDs reached up to 10 g/L because PEI-CdS QDs are water-soluble and can be well dispersed in precursor solutions, as well as in the hydrogel film.

**Figure 5:**
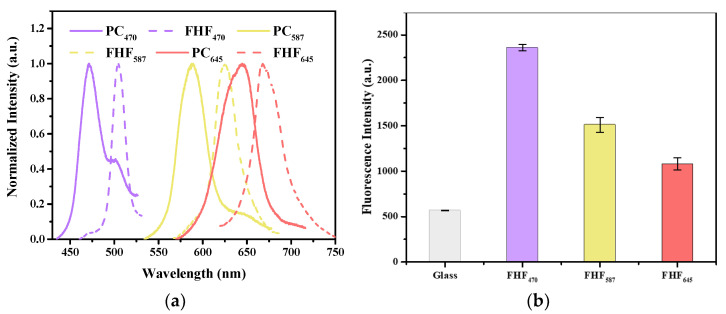
Reflectance spectra of photonic crystals and their corresponding fluorescent photonic crystal hydrogel films (**a**) and comparison of the maximum fluorescence intensity of the control film and the prepared hydrogel films with different stopbands of photonic crystal templates (**b**).

Subsequently, the influence of the photonic crystal structure on the fluorescence emission property of the PEI-CdS QD-embedded photonic crystal hydrogel was studied. We prepared photonic crystal templates with different stopbands located at 470 nm, 587 nm, and 645 nm (named PC_470_, PC_587_ and PC_645_) by using polystyrene microspheres with different particle sizes (Figure 5a, solid lines). After hydrogel formation, the stopband of the film showed a slight red shifting (Figure 5a, dot lines), owing to the increase in the effective refractive index of the hydrogel film. The corresponding fluorescent photonic crystal hydrogel films were named FHF_470_, FHF_587_, and FHF_645_. The control hydrogel film without a photonic crystal structure was also prepared and named Glass. Figure 5b displays the maximum fluorescence emission intensity of these four kinds of hydrogel films. It can be seen that the fluorescence intensity of the hydrogel with a photonic crystal structure is much stronger than the control sample, and FHF_470_ shows the maximum fluorescence emission intensity. We ascribe this fluorescence-enhancement effect to the special stopband propagation behavior resulting from the photonic crystal structure. It has been reported that the propagation of light in a certain wavelength range is prohibited in photonic crystal structures, while the group velocity of photons tends to zero at the edge of the stopband, increasing the time of reaction between quantum dots and photons, resulting in fluorescence enhancement [37,38,39]. For FHF_470_, a slow photon effect was produced, owing to the obvious overlap between its emission band and stopband; its fluorescence intensity thus increased significantly, whereas FHF_587_ and FHF_645_ have fewer overlapping areas between their emission bands and stopbands, so the fluorescence enhancement effect is weaker. These results indicate that the introduction of photonic crystal structures into the fluorescent hydrogel is an effective method for enhancing fluorescence intensity.

**Figure 6:**
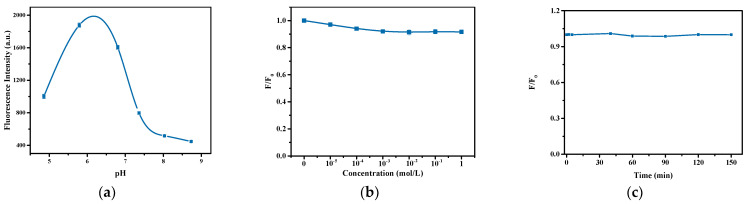
Effect of pH (**a**), ionic strength (**b**), and exposure time under UV light (**c**) on the fluorescence stability of FHF_470_.

Fluorescence stability is critical in the application of fluorescent materials. The effect of pH, ionic strength (NaCl concentration), and exposure time of UV light on the fluorescence stability of the obtained fluorescent photonic crystal hydrogel were studied. As shown in Figure 6a, the experimental results show that the maximum fluorescence intensity of photonic crystal hydrogel was achieved at pH 6. As illustrated in Figure 6b,c, compared with the hydrogel in aqueous solution, the fluorescence intensity was almost unchanged when the NaCl concentration increased from 10^−5^ M to 1 M, or under the continuous irradiation of UV light for 150 min. These results indicate that the as-prepared hydrogel has good optical stability against ionic strength and UV light, which is beneficial for sensing applications.

### 3.3. Sensing Application of the Hydrogel

**Figure 7:**
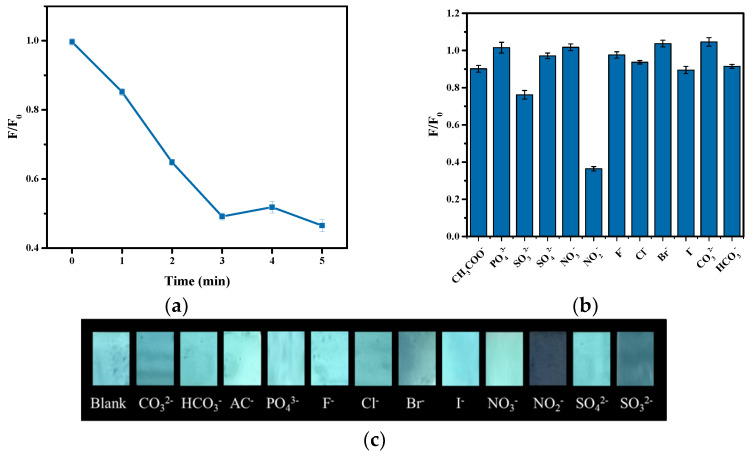
The fluorescence changing of the hydrogel after incubating with NO_2_^−^ for different times (**a**), with different ions (**b**), and the fluorescence images of the gel after interacting with different ions (**c**).

In our previous work, it was found that PEI-CdS QDs could work as an excellent fluorescence sensor for the detection of nitrite in water [36]. Thus, the fluorescence-sensing performance of the as-prepared PEI-CdS QD-embedded photonic crystal hydrogel for the detection of nitrite was tested. The hydrogel was immersed in nitrite aqueous solution (1 × 10^−3^ M) for certain amounts of time, and its fluorescence intensity was measured. As displayed in Figure 7a, the fluorescence intensity of the hydrogel decreases significantly in the first three minutes and then stays flat. This means that nitrite can react with QDs in the hydrogel quickly and quench its fluorescence efficiently. It is worth noting that the fluorescence intensity begins to reduce within 30 s; the reaction is rapid. It is believed that the 3D network structure of the hydrogel improves mass transfer, and it does not take long for nitrite to react with PEI-CdS QDs and produce fluorescence quenching. The effect of different ions, including NO_2_^−^, NO_3_^−^, CO_3_^−^, HCO_3_^−^, SO_4_^2−^, SO_3_^2−^, CH_3_COO^−^, PO_4_^3−^, Cl^−^, Br^−^, I^−^, and S^2−^ on the fluorescence of the hydrogel were also evaluated (Figure 7b). It is clear that most of the above-mentioned ions only exhibit a weak quenching effect on the fluorescence intensity of the hydrogel, and their quench degrees are much lower than NO_2_^−^. This indicates nitrite can be specifically detected by the hydrogel. Figure 7c shows fluorescence images of the PEI-CdS QD-embedded photonic crystal hydrogel after incubating with different ions. It is observed that nitrite has a strong quenching effect on the fluorescence intensity, and this result is in good accordance with that in Figure 7b. The above experimental results show that the as-prepared PEI-CdS QD-embedded photonic crystal hydrogel can be applied as a fluorescence sensor for the detection of nitrite.

**Figure 8:**
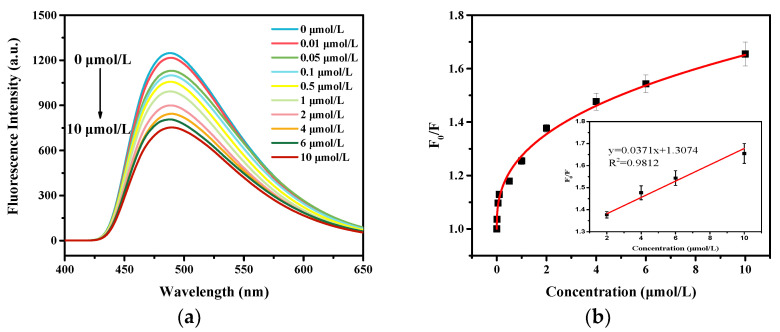
Fluorescence spectra of the fluorescence sensor in the presence of different concentrations of NO_2_^−^ (**a**) and the plot of F_0_/F versus NO_2_^−^ concentration (**b**).

The sensitivity of this fluorescence sensor was then estimated with different concentrations of nitrite in a range from 0 μmol/L to 10 μmol/L. It can be seen from Figure 8a that the fluorescence intensity of the hydrogel decreases with the increase in NO_2_^−^ concentration, indicating that the hydrogel is sensitive to nitrite. The quantitative relationship in Figure 8b indicates that there is a good linear relationship (R^2^ = 0.981) between F_0_/F and the nitrite concentration from 2.0 μmol/L to 10 μmol/L. The limit of detection of this sensing platform was measured to be 0.25 μmol/L, which is much lower than the maximum limit of nitrite in drinking water (65 μmol/L), as ruled by the World Health Organization.

**Figure 9:**
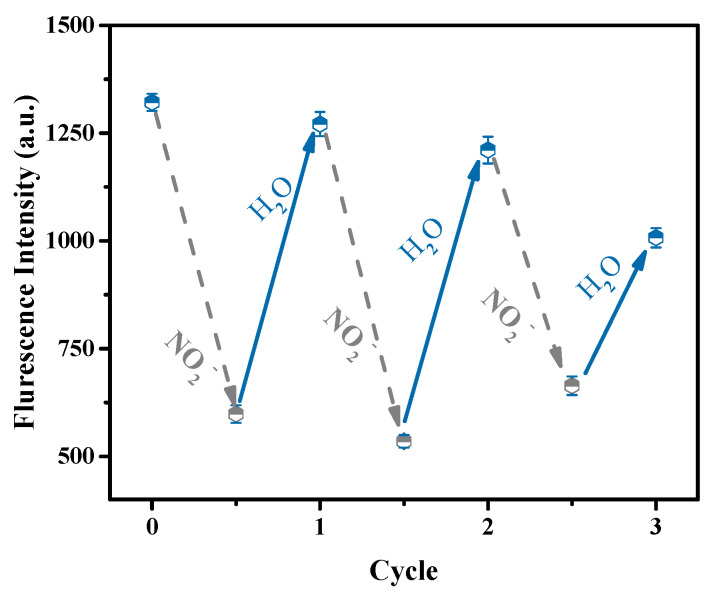
Fluorescence intensity of fluorescence sensor in three cycles.

After the sensing process, the fluorescence sensor can easily be separated from the nitrite aqueous solution just by removing it by hand. Furthermore, its fluorescence can be recovered by rinsing it with pure water. Therefore, the reusability of the fluorescence sensor was studied. The fluorescence sensor was alternately immersed in nitrite aqueous solution and pure water three times, and the fluorescence intensity was measured and is shown in Figure 9. It can be seen that during the first two cycles, the fluorescence quenching and the fluorescence intensity are almost completely repeatable. After three cycles, the fluorescence can be partially restored. These results illustrate that this sensing platform is reusable to a certain extent. It is significant to be able to use the hydrogel repeatedly, and it is confirmed to be operable as a reusable sensor for the detection of nitrite.

## 4. Conclusions

In this contribution to the research, a novel PEI-CdS QD-embedded fluorescent photonic crystal hydrogel was fabricated. PEI-CdS QDs can be well dispersed in the hydrogel and endow the hydrogel with photoluminescence capability. More importantly, the photonic crystal structure can enhance the fluorescence intensity of the hydrogel efficiently. This fluorescence-enhancement effect was ascribed to the special propagation behavior of a stopband in photonic crystals. The fluorescence of the hydrogel was quite stable, even under high ionic strength conditions or under the continuous irradiation of UV light. In addition, the as-prepared fluorescent photonic crystal hydrogel can be used as a reusable fluorescence sensor for the detection of nitrite in water with good sensitivity and selectivity. This study provides a novel fluorescence-enhancement strategy and is encouraging for the development of hydrogels to be used as fluorescent-sensing platforms.
